# Dr. John M. Riggs

**Published:** 1885-12

**Authors:** 


Dr. John M. Riggs.—Died at Hartford, Conn., Nov. 11th, 1885, of typhoid pneumonia in the seventy-sixth year of his age. Dr. Riggs was one of the old-school practitioners of dentistry, having commenced practice in 1840 in Hartford, where he continued to reside to the date of his death. Two years before the anæsthetic use of ether, in the year 1844, Dr. Riggs extracted a tooth for Dr. Horace Wells, while the patient was under the influence of nitrous oxide gas. Dr.

Riggs never married, but was a greatly esteemed citizen, and a leading practitioner of dentistry, gaining considerable reputation for a heroic method of treating alveolar pyorrhœa, this affection being commonly called "Riggs' Disease." Dr. Riggs was one of the vice-presidents of the Southern Dental Association at the time of his death, having been elected to that position at the New Orleans meeting of 1885.

